# Consistent Richness-Biomass Relationship across Environmental Gradients in a Marine Macroalgal-Dominated Subtidal Community on the Western Antarctic Peninsula

**DOI:** 10.1371/journal.pone.0138582

**Published:** 2015-09-18

**Authors:** Nelson Valdivia, María José Díaz, Ignacio Garrido, Iván Gómez

**Affiliations:** Instituto de Ciencias Marinas y Limnológicas, Facultad de Ciencias, Universidad Austral de Chile, Valdivia, Chile; Central Michigan University, UNITED STATES

## Abstract

Biodiversity loss has spurred the biodiversity-ecosystem functioning research over a range of ecosystems. In Antarctica, however, the relationship of taxonomic and functional diversity with ecosystem properties (e.g., community biomass) has received less attention, despite the presence of sharp and dynamic environmental stress gradients that might modulate these properties. Here, we investigated whether the richness-biomass relationship in macrobenthic subtidal communities is still apparent after accounting for environmental stress gradients in Fildes Bay, King George Island, Antarctica. Measurements of biomass of mobile and sessile macrobenthic taxa were conducted in the austral summer 2013/4 across two environmental stress gradients: distance from nearest glaciers and subtidal depth (from 5 to 30 m). In general, community biomass increased with distance from glaciers and water depth. However, generalised additive models showed that distance from glaciers and depth accounted for negligible proportions of variation in the number of functional groups (i.e., functional richness) and community biomass when compared to taxonomic richness. Functional richness and community biomass were positive and saturating functions of taxonomic richness. Large endemic, canopy-forming brown algae of the order Desmarestiales dominated the community biomass across both gradients. Accordingly, differences in the composition of taxa accounted for a significant and large proportion (51%) of variation in community biomass in comparison with functional richness (10%). Our results suggest that the environmental factors here analysed may be less important than biodiversity in shaping mesoscale (several km) biomass patterns in this Antarctic system. We suggest that further manipulative, hypothesis-driven research should address the role of biodiversity and species’ functional traits in the responses of Antarctic subtidal communities to environmental variation.

## Introduction

Climate-change effects on natural communities and human activities can be already observed worldwide. A direct consequence of climate change is the local alteration of natural populations (reviewed in [1]), and a recent major synthesis indicates that the ecosystem-level consequences of species loss can be comparable to those of global-change stressors like ultraviolet radiation, climate warming, and elevated CO_2_ [2]. The marine Antarctic ecosystems show probably the world’s fastest responses to climate change, with glacier retreat being an important source of change in environmental stress regimes [3,4]. These changes in stress—i.e. the environmental forcing that negatively affects the performance of organisms [5,6]—are rapidly affecting the abundance of Antarctic species (e.g. [7]). Recent surveys point to the need for intensification of studies on the projected impact of global change-driven processes on benthic communities in order to predict further consequences for marine functioning (e.g. [4,8]). Despite these rapid environmental changes, the biodiversity-ecosystem functioning research in Antarctica has lagged behind in development compared to systems from other regions.

Biodiversity can be described in terms of the number of taxonomic entities (i.e. species or taxon richness), differences in their functional traits, and their interactions [9]. The relationship between taxonomic and functional richness determines how biodiversity modulates ecosystem properties (e.g. community biomass) and partly depends on the mechanisms of community assembly (reviewed in [10]). For example, niche differentiation implies that the functional characteristics of species should vary in order to allow coexistence, leading to a positive relationship between the number of taxa and the number of functional groups [11]. These positive and saturating responses of functional richness to taxonomic richness indicate that local diversity ensures the provision of functional traits [12]. However, the physical environment can have stronger effects on some species than others; this, in turn, limits the range of functional traits in the assemblage, so that increasing taxonomic richness may not lead necessarily to an increased diversity of functional responses to the environment [11]. Accordingly, harsh environmental conditions can weaken the relationship between taxonomic and functional richness [13].

Positive richness-biomass relationships can arise from the combination of, at least, two processes. First, species-rich communities are more likely to contain highly productive species (i.e. “selection probability” or “compositional” effects [14]). Second, increased taxonomic richness can lead to more productive communities through positive species interactions, such as differential use of resources (i.e. resource complementarity through niche partitioning) and facilitation. Albeit manipulative experiments are useful and desirable to tease apart the contribution of both processes to the richness-biomass relationship, they face difficulties to account for broad-scale patterns of biomass along environmental gradients. Alternatively, variation-partition techniques (e.g. [15]) would provide relevant information from observational richness-biomass patterns in order to construct hypotheses regarding the roles of compositional and resource complementarity effects in a given region.

**Fig 1:**
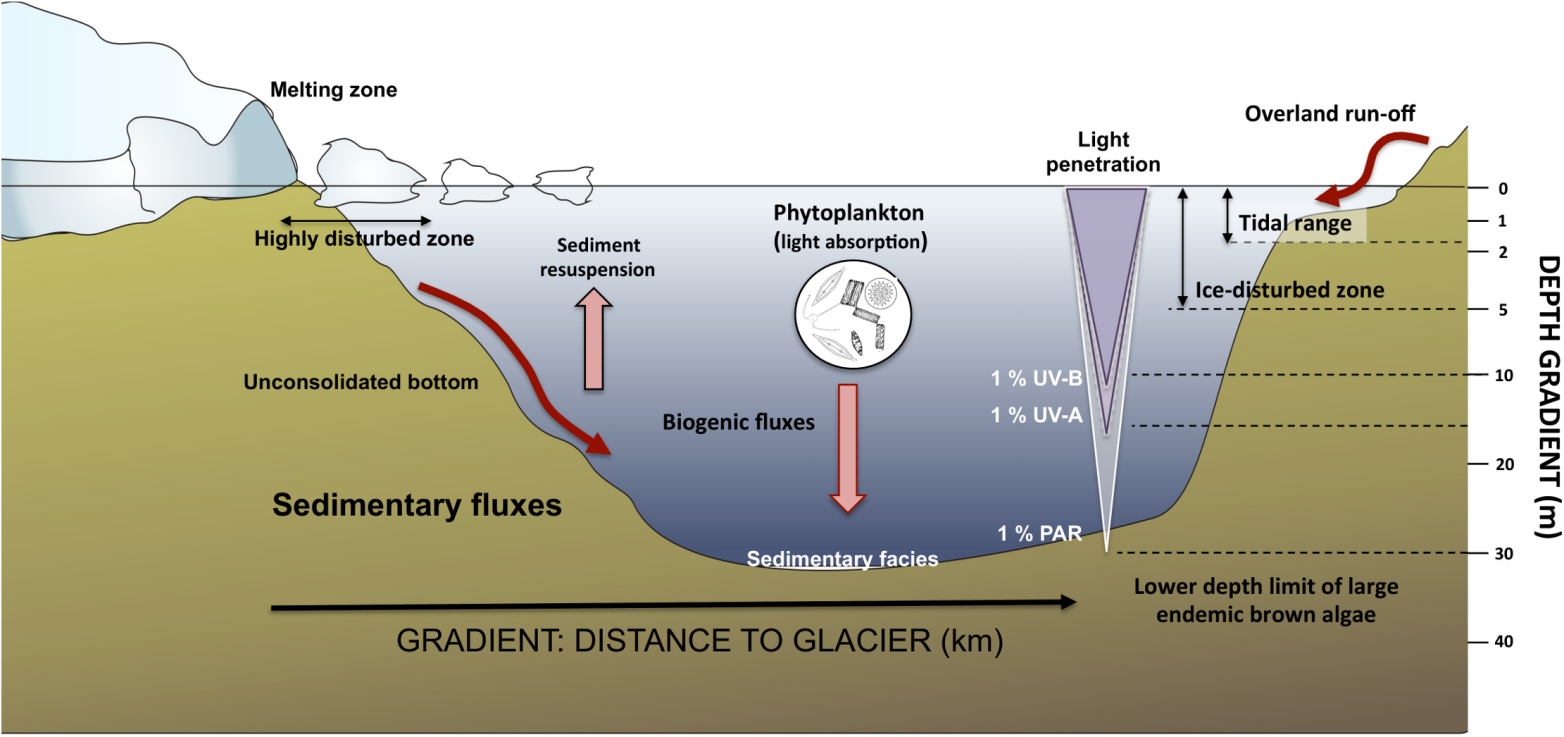
Environmental gradients in Fildes Bay defined by the distance to the surrounding glaciers (in scales of kilometers) and depth (in scale of meters). Both gradients are strongly modulated by light penetration and sedimentation processes caused by glacier dynamics. Depth penetration of photosynthetically active radiation (PAR) and UV radiation are indicated.

Observational work has shown variable richness-biomass relationships in natural communities [10,16–18]. An explanation to these patterns is that the relationships between taxonomic or functional richness and community biomass vary along environmental stress gradients [13,18,19]. In Antarctic coastal ecosystems, distance to glaciers and depth define two major environmental gradients in terms of mechanical disturbances and physiological stress (see Fig 1 and refs. [20–23]). The closeness to glaciers is related to harsh environmental conditions for macrobenthic assemblages due to mechanical disturbance by ice scours and physiological stress by enhanced turbidity and sedimentation (e.g. [23]). Accordingly, significant differences in subtidal macroalgal abundances can be observed within a couple of kilometres from melting glaciers [7]. Regarding water depth, sharp vertical gradients in light availability and seasonal impact of ice scouring on shallow-water assemblages generate a vertical disturbance-stress gradient that modulates the distribution of dominant macroalgae [22,24–27]. Accordingly, we might hypothesise that harsh environmental conditions due to glacier proximity and shallow water restrict the diversity of functional traits, which in turn might lead to weak richness-biomass relationships of Antarctic macrobenthic communities.

Hereby, we explored the relationship between the number of taxa, functional groups richness, and environmental disturbance-stress gradients (distance to nearest glaciers and depth, hereafter referred to as stress gradients) with macrobenthic community biomass in a subtidal Antarctic system of King George Island, South Shetlands. The number of functional groups (i.e. functional richness) was partitioned into the number of functional traits that determine organisms’ responses to environmental changes (i.e. functional *response* traits), and those that determine organisms’ effects on ecosystem properties (i.e. functional *effect* traits). We first tested whether, after accounting for the effects of environmental stress gradients, functional richness and its components are positive and saturating functions of the number of taxa (taxonomic richness). Then, we tested whether community biomass significantly fit to taxonomic richness in addition to the environmental gradients. Finally, we used variation-partition analyses to infer the relative contributions of compositional effects and resource complementarity to the richness-biomass relationship.

## Materials and Methods

### Ethic statement

This work was conducted as part of the activities carried out by the Algas Antárticas working group at the Universidad Austral de Chile and fully approved by Dr. José Retamales, director of the Instituto Antártico Chileno (INACH), in accordance with the Protocol on Environmental Protection to the Antarctic Treaty. No additional specific permissions were required for any sampling site, as they were located outside of the Antarctic Specially Protected Areas and the study did not involve endangered or protected species.

### Study region

The study was conducted in Fildes Bay, King George Island (Fig 1), during the austral summer 2014 (January-February). The 14 km long and 6–14 km wide Fildes Bay regularly freezes in austral winter, from late July to mid-September [28–30]. In this location, the subtidal assemblages between 10 and 30 m are characterised by brown algae such as *Himantothallus grandifolius* and *Desmarestia anceps*, and red algae such as *Trematocarpus antarcticus*, *Plocamium cartilagineum*, and *Palmaria decipiens* [27]. The grazer gastropods *Nacella polaris*, *Margarella* sp., the sea urchin *Sterechinus neumayerii*, and the predatory sea stars *Odontaster validus* and *Diplasterias brucei* are the most conspicuous benthic mobile invertebrates [28,30]. Sessile invertebrates such as bryozoans, ascidians, and sponges are also abundant [31,32].

In the study area, we located six sites at increasing distances (from 100s to 1000s of metres) from glaciers Nelson and Collins (Table 1). For each site, we conduced underwater irradiance measurements in order to represent the variation in a relevant environmental factor for the studied assemblages, which are largely dominated by photosynthetic organisms (see the previous paragraph above) that perform significant and species-specific responses to variations in solar irradiation in Fildes Bay [27]. Vertical profiles of underwater solar radiation were determined by means of a Biospherical 2500 radiometer (Biospherical Inc., San Diego, USA). The measurements were conducted during solar noon at each site, with the exception of Nelson Strait, which could not be sampled due to logistic constraints. Light measurements were restricted to sunny days with less than 20% of cloud coverage. Due to the low, and sometimes unpredictable, number of sunny days at Fildes Bay during the study period, the sample size for each location remained relatively low (n = 2 to 4). In order to describe how irradiance attenuates with depth, we used the following equation: $E_{d}(Z)=E_{d}(0)e^{-K_{d}z}$, where *K*_*d*_ is the vertical attenuation coefficient for irradiance, *E*_*d*_ (*Z*) is the irradiance at depth *z*, and *E*_*d*_ (0) is the irradiance just below the surface [33]. The slope from the linear regression between the ln of irradiance *E*_*d*_ (*Z*) and depth *z* was used to estimate the vertical attenuation coefficients (*K*_*d*_) for 305 nm (UVB), 340 nm (UVA), and the range between 400–700 nm (photosynthetically active radiation, PAR).

**Table 1:** List of sampling sites surveyed in this study and their coefficients of vertical irradiance attenuation (*K*_*d*_ m^-1^). Latitude and longitude are expressed as decimal degrees. *K*_*d*_ m^-1^ values are expressed as means (n = 2 to 4) and standard errors of the means (bracketed). NA stands for “not available”.

	Site name	Code	Latitude	Longitude	Vertical attenuation coefficients (*K*_*d*_ m^-1^)		
					UVB	UVA	PAR
1	Collins Glacier	CG	-62.16	-58.84	0.49 (0.02)	0.29 (0.02)	0.16 (0.01)
2	Artigas	AT	-62.18	-58.87	0.49 (0.01)	0.35 (0.01)	0.19 (0.03)
3	Fildes	FD	-62.20	-58.94	0.35 (0.01)	0.22 (0.01)	0.12 (0.01)
4	Ardley Peninsula	PA	-62.22	-58.94	0.42 (0.01)	0.24 (<0.01)	0.12 (0.01)
5	Nelson Strait	NS	-62.24	-58.97	NA	NA	NA
6	Nelson Glacier	NG	-62.26	-58.98	0.53 (0.03)	0.26 (0.03)	0.14 (0.01)

### Sampling

At each site we haphazardly located five 50 x 50 cm plots at 5–10, 15–20, and 25–30 metres depths by means of SCUBA diving. The plots were separated ca. 10 metres from each other. For each plot, we removed all sessile and mobile macrobenthic (>5 mm length) organisms, which corresponded to our lowest detection limit in situ. All the sampled organisms from a given plot were placed and transported in a 5-mm-pore individual mesh bag. Sessile organisms were removed from the substratum with a scraper. Special care was placed in sampling mobile organisms that were associated to macroalgae. However, very mobile organisms (e.g. amphipods and small substrate-associated gastropods [34]) could well have escaped from the sample and probably their values of biomass were underestimated. Since large organisms dominate these assemblages (see *Study region* section above), nevertheless, missing small albeit ecologically relevant organisms should have had a limited impact on the estimation of functional group and community biomass.

In the laboratory, organisms were sorted and identified to the lowest taxonomic level possible, usually to species level. Morphological and life history traits of taxa were reviewed in references [35–38]. Each organism was weighted (wet weight, 0.01 g accuracy, here after referred to as “ww”), and small individuals such as amphipods were weighted in groups in order to estimate average individual weight.

Species’ traits were defined as those characteristics that determine how the species responds to environmental changes (i.e. functional *response* traits) or how the species affects community biomass (i.e. functional *effect* traits; see also refs. [1,10]). Each taxon was categorised according to two response traits, i.e. mobility and dispersal potential; and three effect traits, i.e. trophic type, growth type, adult body size (Table 2). We assigned to each taxon a 5-character code according to all possible combinations of traits (see categories in Table 2). Then, the biomass of each code (i.e. functional trait group) was estimated by summing the biomass of all taxa for each group [39,40]. Finally, the number of taxonomic identities and functional trait groups (hereafter referred to as taxon richness and total functional richness, respectively), the biomass of each functional trait group (i.e. response and effect richness), and community biomass were estimated from the taxon biomass dataset. Response and effect richness were analysed separately.

**Table 2:** Functional response and effect traits used to categorise the subtidal taxa identified in Fildes Bay during the austral summer 2014. Each taxon was scored with a combination of uppercases (bracketed) in order to estimate functional richness and biomass.

Functional response traits		Functional effect traits		
Mobility	Dispersal potential	Trophic type	Growth type	Adult body size
Sessile (S)	No planktonic stage (N)	Autotrophs (A)	Bushy (B)	*<*1 cm (S)
Mobile (M)	Anchiplanic (A) ^1^	Deposit feeder (D)	Filamentous (F)	1–10 cm (M)
Limited mobility (L)	Actaeplanic (C) ^2^	Herbivores (H)	Encrusting (E)	10–100 cm (L)
		Carnivores (C)	Massive (M)	100–1000 cm (X)
		Suspension feeder (S)	Foliose (L)	>1000 cm (G)

^**1**^ Anchiplanic are those organisms with stages that stay few hours to a few days in the plankton

^**2**^ Actaeplanic organisms stay one week to two months in the plankton.

### Statistical analysis

Generalised additive models (GAMs [41]) were used to analyse the richness, biomass, and spatial data. The flexibility of GAM allows assessing non-linear relationships without fitting arbitrarily selected functions [42]. Therefore, we considered GAM as a suitable tool for examination of saturating response of ecosystem properties to taxonomic richness. We fit (1) total functional richness, (2) functional response richness, (3) functional effect richness, and (4) log_10_-transformed community biomass in separate models as response variables. The models included as explanatory variables the taxonomic richness, the linear distance to nearest glacier (km), and depth stratum (three levels: 5–10, 15–20, and 25–30 m). In order to account for the contribution of taxon composition to community biomass patterns, we included in the model the PC1 and PC2 from a principal component analysis on the log_10_-transformed matrix of taxon biomass. We assumed a Poisson and Gaussian distribution of errors for functional richness (fits 1 to 3) and community biomass (fit 4), respectively. The appropriate smoothness for each model was found by means of Un-Biased Risk Estimators (UBRE) and Generalised Cross Validation (GCV) for functional richness and biomass, respectively. Model selection was done according to automatic penalisation and additional shrinkage of the smooth terms to zero, so that uninformative terms were removed from the model. In addition, we used interactive model selection in which each term was checked and removed when three criteria were fulfilled: (i) The estimated degrees of freedom were close to zero (due to shrinkage of smooth term), (ii) the confidence intervals for the smooth everywhere included zero, and (iii) the UBRE or GCV scores decreased after the term is removed from the model [43,44].

Finally, we partitioned the variation in community biomass with respect of total functional richness and the matrix of taxonomic identity biomass (i.e. taxonomic composition) in order to assess the degree to which resource complementarity and compositional effects could have influenced the richness-biomass relationship. We used adjusted *r* ^2^ in redundancy analysis ordinations (RDA) as estimators of each conditioned fractions of variance. The significance of variance fractions (functional richness and taxonomic composition) was tested by means of a RDA [15,45]. GAMs and variance partitioning were conducted in the mgcv and vegan packages of the R environment version 3.1.1, respectively [46].

## Results

### Environmental variability in terms of vertical irradiance attenuation

Vertical irradiance attenuation (*K*_*d*_ m^-1^) showed the minimum values at sites located in the centre of the bay, in particular Fildes (Table 1). For the UVB wavelength, the maximum values of attenuation were observed near both glaciers (Collins and Nelson) and Artigas. On the other hand, the attenuation of UVA and PAR peaked at the Artigas, followed by Collins and Nelson glaciers (Table 1).

### Spatial variability of taxonomic richness and functional groups biomass

**Fig 2:**
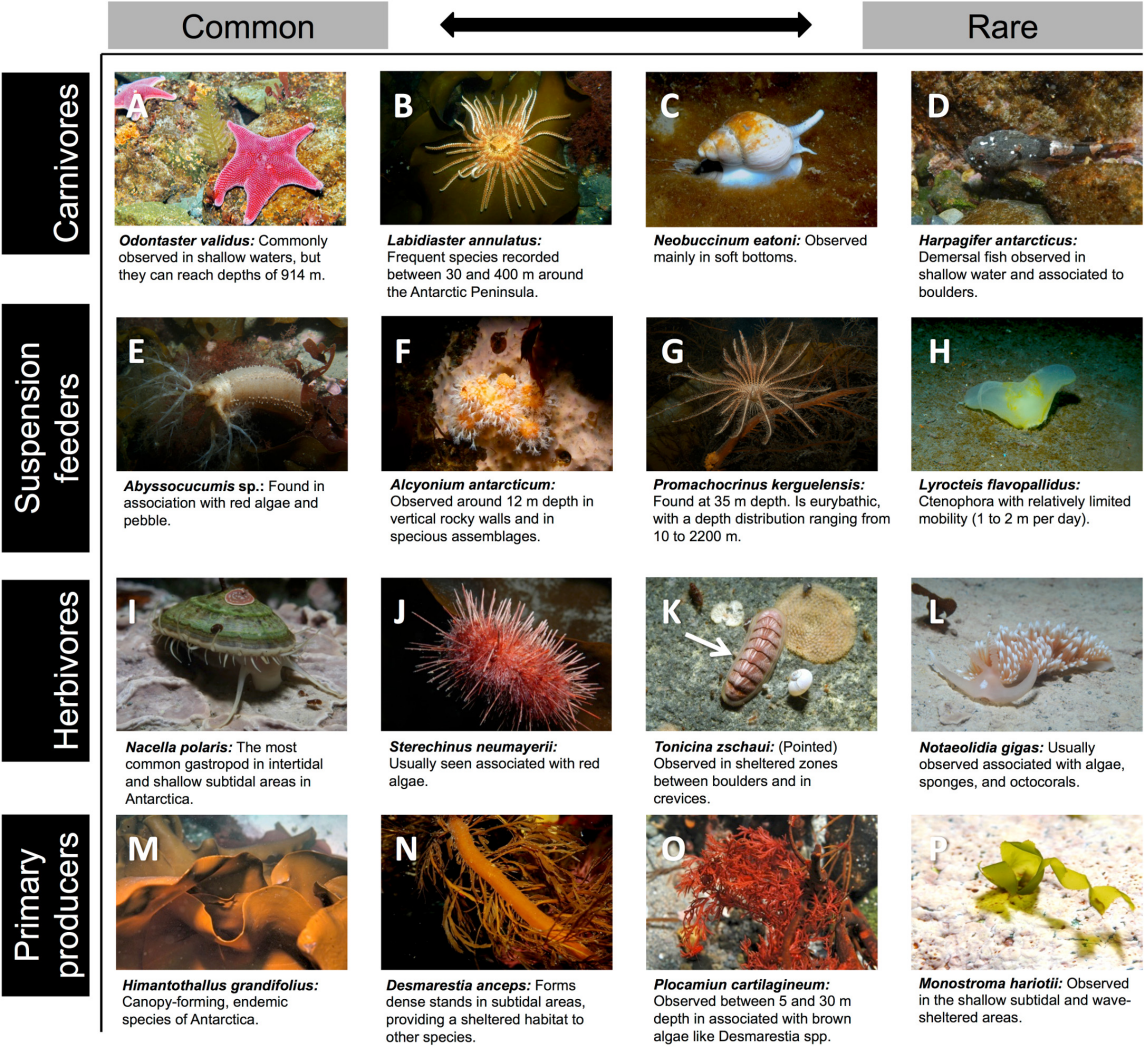
Representative taxa identified in Fildes Bay. Taxa are sorted according to trophic level (rows) and frequency of occurrence (columns). Common and rare taxa occurred on >80% and <10% of the sampling units, respectively. Remarks for each taxon are based on authors’ personal observations and published data [35–38].

**Fig 3:**
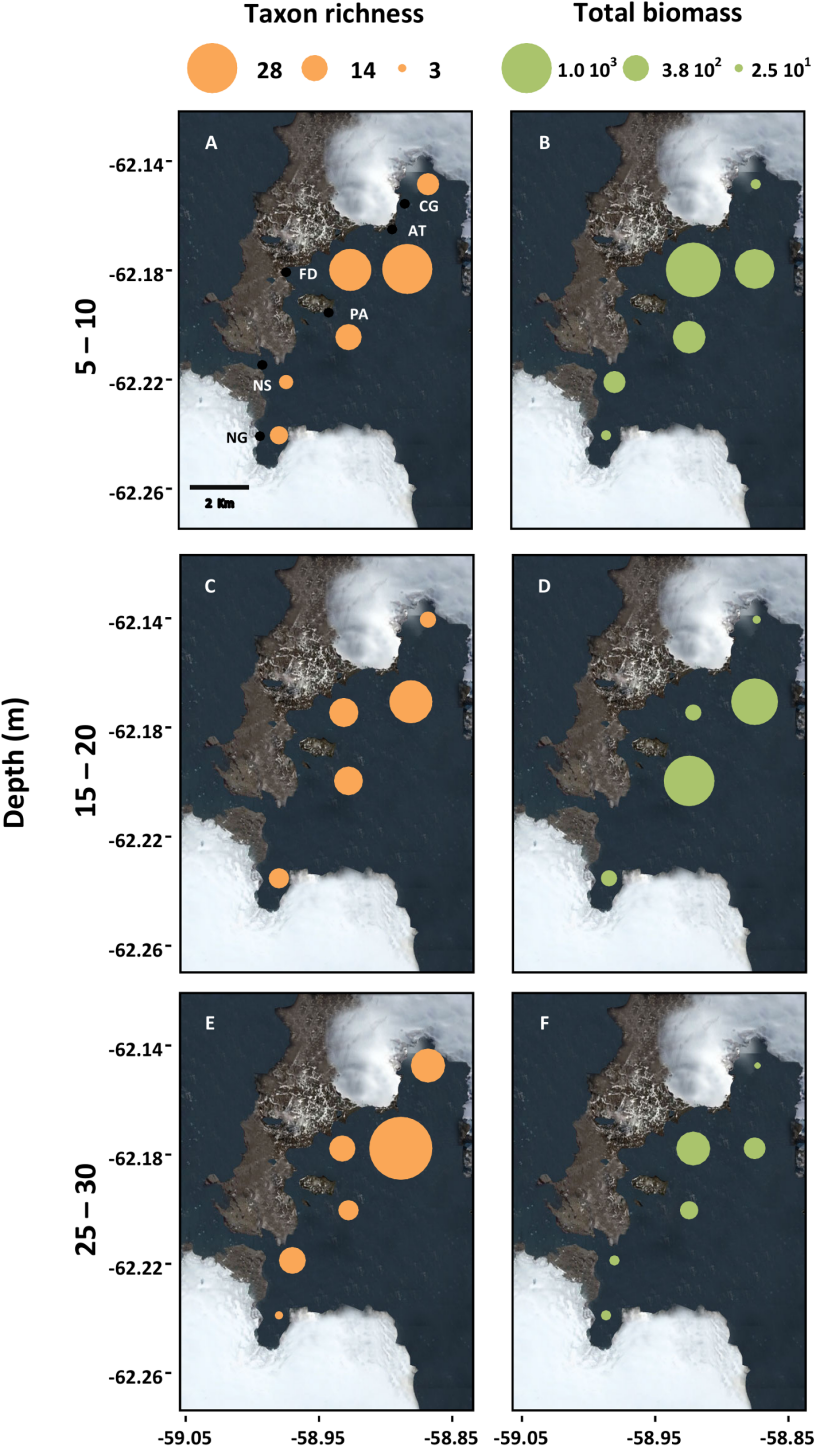
Spatial patterns of site-specific taxon richness and community biomass in Fildes Bay. Site codes are in Table 1.

A total of 66 taxonomic identities (Table 3), accounting for a mean community biomass of 1445 g m^-2^ ww, were identified in the study area. The benthic system was characterised by taxa varying from very abundant and ubiquitous—such as the sea star *O*. *validus* (Fig 2A), the suspension-feeding sea cucumber *Abyssocucumis* sp. (Fig 2E), the gastropod *N*. *polaris* (Fig 2I), and the brown alga *H*. *grandifolius* (Fig 2M)—to taxa with rare occurrence and very low biomass, e.g. the shallow-water green alga *Monostroma hariotii* (Fig 2P). Site-level taxonomic richness ranged from three to 28 identities and community biomass from 417 g m^-2^ ww to 2627 g m^-2^ ww (Fig 3). Taxon richness and community biomass showed a high spatial variation, with higher values in sites located near the centre of the bay (Fig 3). In addition, the highest taxonomic richness was observed at 25–30 m depth, while total biomass was highest between 5–15 m (Fig 3).

**Table 3:** List of taxonomic identities identified at six subtidal sites at Fildes Bay, King George Island. The occurrence of each taxon in each site is shown.

Taxon	Collins Glacier	Artigas	Fildes	Ardley Peninsula	Nelson Strait	Nelson Glacier
**Chlorophyta**						
*Monostroma hariotii*		x	x	x		
**Ochrophyta**						
*Ascoseira mirabilis*	x	x		x		
*Cystosphaera jacquinotii*		x				
*Desmarestia anceps*	x	x		x	x	
*Desmarestia antarctica*	x			x	x	
*Desmarestia menziesii*	x	x		x	x	x
*Halopteris obovata*		x		x		
*Himantothallus grandifolius*	x	x	x	x	x	x
**Rodophyta**						
*Antarcticothamnion polysporum*			x			
*Ballia callitricha*		x	x			
*Callophyllis atrosanguinea*		x				
*Callophyllis* sp.		x				
*Callophyllis variegata*		x				
*Ceramium* sp.		x				
*Curdiea racovitzae*		x		x		
*Delesseria* sp.	x		x		x	x
*Georgiella confluens*		x				
*Gigartina skottsbergii*	x		x	x		
*Gymnogongrus* sp.		x				
*Hymenocladiopsis* sp.			x			
*Iridaea cordata*			x		x	
*Myriogramme manginii*			x			
*Pachymenia* sp.		x		x		
*Palmaria decipiens*	x	x		x	x	x
*Pantoneura plocamioides*	x	x		x		
*Phycodrys* sp.	x		x			
*Picconiella plumosa*		x		x		
*Plocamium cartilagineum*		x		x		
*Trematocarpus antarcticus*			x			
**Porifera**						
Unid. Porifera sp. 1	x	x	x	x		
**Cnidaria**						
Unid. Alcyonacea sp. 1		x				
Unid. Hydrozoa sp. 1		x				
**Annelida**						
*Flabelligera mundata*	x	x	x	x	x	
**Mollusca**						
*Laternula elliptica*	x					
*Limea pygmaea*	x	x	x		x	
*Margarella* sp.	x	x	x	x	x	x
*Nacella polaris*	x	x	x	x	x	
*Neobuccinum eatoni*				x	x	x
Unid. Gastropoda sp. 1	x	x				
Unid. Polychaeta sp. 1	x	x	x	x	x	
Unid. Polyplacophora sp. 1			x			
Unidentified Venereidae sp. 1	x				x	
Unidentified Venereidae sp. 2		x				
Unidentified Venereidae sp. 3	x	x				
*Yoldia eightsii*			x			
**Arthropoda**						
*Glyptonotus antarcticus*	x					
Unid. Gammaridae sp. 1	x	x	x	x	x	
Unid. Pycnogonida sp. 1		x		x		
Unid. Serolidae sp. 1		x	x			
**Equinodermata**						
*Abatus agassizii*					x	
*Abyssocucumis* sp.	x	x	x			
*Diplasterias brucei*	x	x	x	x		x
*Odontaster meridionalis*			x	x		
*Odontaster validus*	x	x	x	x	x	x
*Ophionotus* sp.		x	x	x	x	x
*Sterechinus neumayeri*			x			
Unid. Asteroidea sp. 1	x	x	x	x		
Unid. Asteroidea sp. 2					x	
**Chordata**						
*Cnemidocarpa verrucosa*			x			
Unid. Ascideacea sp. 1		x	x	x	x	
**Others**						
*Parborlasia corrugatus*			x			
Unid. Brachiopoda sp. 1		x	x	x		
Unid. Bryozoa sp. 1	x	x	x	x	x	
Unid. Nematoda sp. 1		x			x	
Unid. Nemertea sp. 1	x	x				
Unid. Sipunculidae sp. 1		x	x	x	x	x

**Fig 4:**
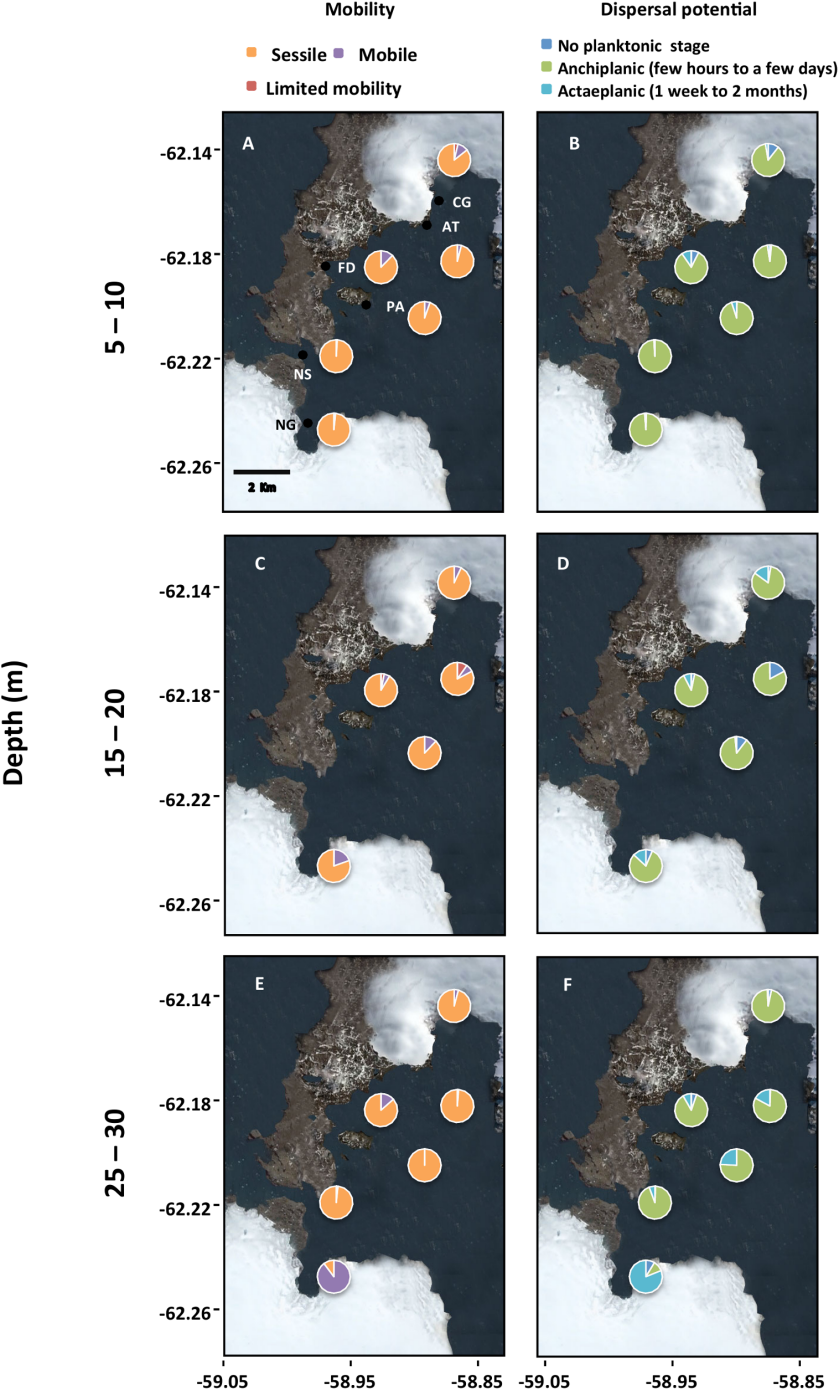
Functional response traits: proportional contributions to site community biomass of taxa grouped according to functional response traits (i.e. mobility and dispersal potential) in the study region. Site codes are in Table 1.

**Fig 5:**
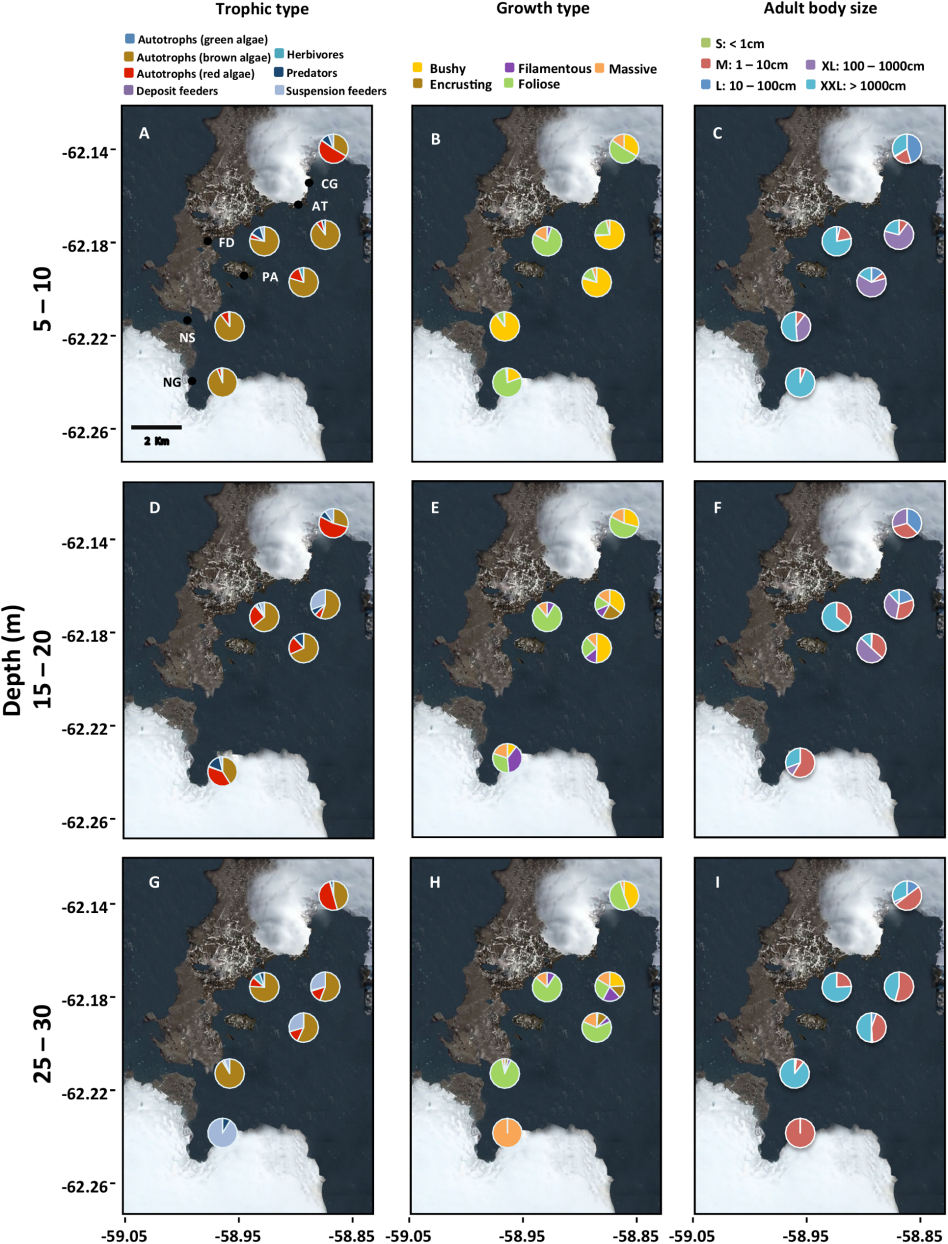
Functional effect traits: Proportional contributions to site community biomass of taxa grouped according to functional effect traits (i.e. trophic type, growth type, and adult body size) in the study region. Site codes are in Table 1.

Regarding species mobility and dispersal potential (i.e. functional response traits), sessile taxa and those with short planktonic stages had the highest contribution to community biomass (Fig 4). This uneven distribution of biomass remained at the three depth strata (Fig 4). In addition, taxa with limited mobility showed a high spatial heterogeneity (coefficients of variation [CV] of 63% and 97% for depth and distance from glacier, respectively). Functional effect traits also showed variable patterns (Fig 5): autotrophs (macroalgae) showed the highest contributions to community biomass, with green algae varying sharply among depth strata and distances from glaciers (CV = ca. 170% for both gradients). On the other hand, brown algae were ubiquitous along both gradients and hence showed lower CV than green algae (CV = 19% and 7% for depth and distance from glacier, respectively). The contribution of other trophic groups was larger at central sites and especially at deeper waters (Fig 5). Suspension feeders dominated the assemblages at Nelson Glacier (Fig 5); this group was more variable along the depth gradient (CV = 89%), than the glacier-distance gradient (CV = 54%). Bushy (e.g. *D*. *anceps*), followed by foliose (e.g. *H*. *grandifolius*), growth forms dominated the shallow-water assemblages (Fig 5B). At deeper waters, the dominance shifted from bushy to foliose forms (Fig 5E and 5F). The highest site-specific number of growth forms was observed at Artigas (AT, centre of the bay). >1000-cm and >100-cm organisms dominated the shallow-water assemblages (Fig 5C); this pattern of dominance shifted toward a >1000-cm and >1-cm pattern at deeper waters (Fig 5G and 5I).

**Fig 6:**
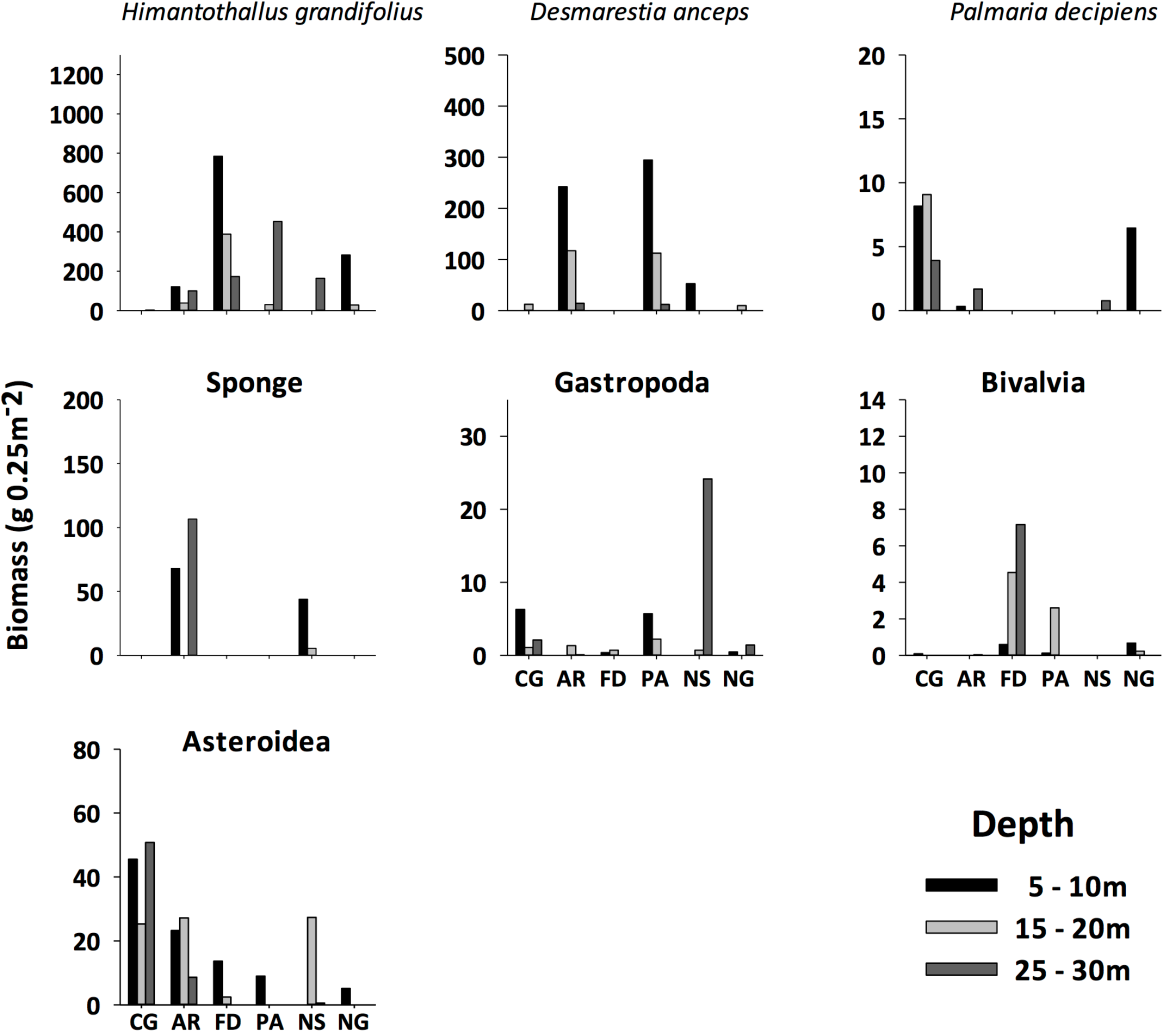
Abundance of major taxonomic groups and species in Fildes Bay. Sites codes are in Table 1. Values are expressed as means ± standard errors of the means.

The spatial patterns of abundances along both environmental gradients strongly varied among major taxonomic groups. The brown alga *Himantothallus grandifolius* showed the highest abundances at central sites (Fig 6A), suggesting that this species accounted for most of the spatial variation of primary producers. *Desmarestia anceps* showed highest abundances at Artigas (AT) and Ardley peninsula (PA, Fig 6B). The abundance of *Palmaria decipiens* peaked at near-glacier sites (Fig 6C). Major invertebrate taxa also varied in relation with the closeness to the glaciers and depth: sponges peaked at AT and Nelson Strait (NS, Fig 6D), Gastropoda also increased at NS (Fig 6E), Bivalvia was more abundance at the centre of the bay (Fig 6F), and Asteroidea, which spread in almost all sites, peaked at Collins Glacier (CG, Fig 6G).

### Relationships of functional richness and community biomass with taxonomic richness

**Fig 7:**
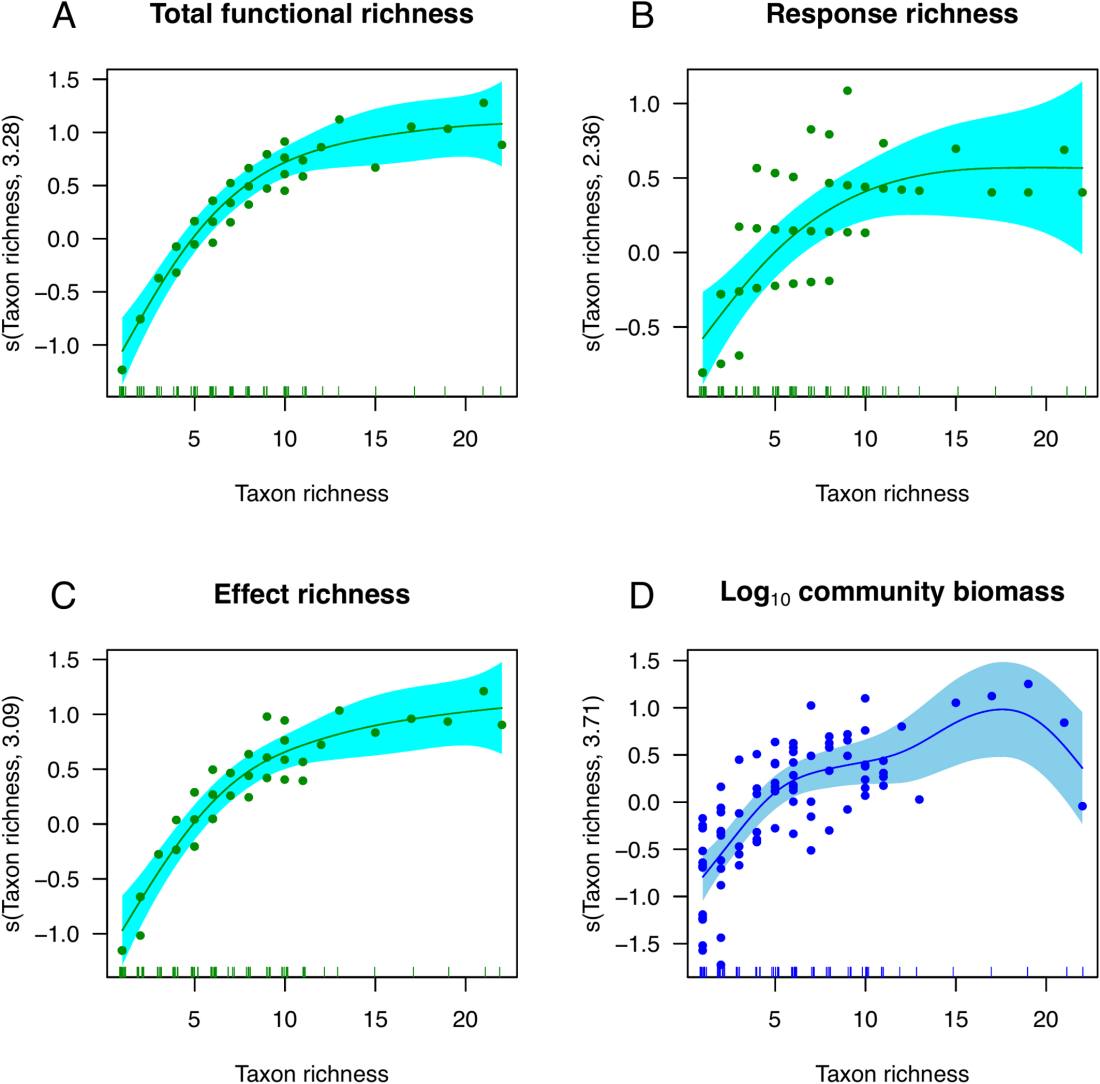
Taxon richness: Estimated generalised additive model (GAM) terms showing the partial residuals (solid dots) and estimated smooth curve (solid line) of the number of all functional trait combinations (Total functional richness, panel A), response traits (Response richness, panel B), effect traits (Effect richness, panel C), and log_10_ transformed community biomass (D). Shaded areas are ± confidence intervals above and below the estimated smooth curve. The estimated degrees of freedom of the plotted terms are in the Y-axis label. Zero values in the Y-axis indicate no effect of taxon richness on the smoothed dependent variable.

According to the model selection procedures, only the smoothed term of taxonomic richness was retained as explanatory variable for three measurements of functional richness analysed (Table 4). The best model for log_10_ community biomass retained all terms except the distance from nearest glacier (Table 4). Log_10_ community biomass tended to decrease with depth, as shown by the negative estimate coefficients (Table 4). The three measures of functional richness showed positive and saturating relationships with taxonomic richness (Fig 7A to 7C), suggesting a high degree of redundancy in the assemblage. According to the distribution of smooth term residuals, log_10_ community biomass was positively related with taxonomic richness, conforming to the expected saturating curve (Fig 7D).

**Table 4:** Estimate parameters of Generalised Additive Models (GAMs) for total functional richness, response and effect richness, and log_10_ transformed community biomass. The full models included depth stratum (Depth, three categories), and the smooth terms of taxon richness [s(TR)], the PC1 and PC2 from a PCA on taxonomic composition [s(PC1) and s(PC2), respectively], and distance to nearest glacier [s(DNG)]. The best models were selected according on extra penalisation of estimated degrees of freedoms of the smooth terms (edf) and interactive model selection. The UBRE and GCV scores, and % deviance explained by each model (Dev.) are provided.

Response		Parametric coefficients			Smooth terms					
	Model	(Intercept)	Depth2	Depth3	s(TR)	s(PC1)	s(PC2)	s(DNG)	Score	Dev.
Total functional richness	Full	1.47	-0.04	-0.02	3.30	~0.00	~0.00	~0.00	0.04	95.5
	Best	1.45			3.28				<0.01	95.3
Response richness	Full	0.88	0.15	0.08	2.28	~0.00	~0.00	~0.00	-0.66	65.4
	Best	0.95			2.36				-0.70	64.5
Effect richness	Full	1.36	-0.11	0.03	3.09	~0.00	~0.00	~0.00	-0.75	93.5
	Best	1.35			3.09				-0.78	92.4
Comm. biomass (log_10_)	Full	2.42	-0.27	-0.35	3.71	2.88	5.50	~0.00	0.24	70.1
	Best	2.42	-0.27	-0.35	3.71	2.88	5.50		0.23	70.1

Both, functional richness and taxonomic composition accounted for significant proportions of variance in log_10_ community biomass (P < 0.01 for both fractions, residual *r* ^2^ = 0.14). Nevertheless, the individual fraction of taxonomic composition was five-fold larger than that of functional richness (adjusted *r* ^2^ = 0.51 and 0.10, respectively). Albeit not testable, the interaction between both sources of variability accounted for a comparatively large fraction of variation in community biomass (adjusted r^2^ = 0.25).

## Discussion

Our results showed that taxonomic richness and community biomass of subtidal organisms varied along two environmental gradients, decreasing with closeness to glacier and water depth. In general, vertical irradiance attenuation tended to increase in the nearby of glaciers, hinting for reduced light availability in these areas. Despite these spatial patterns, the environmental gradients accounted for limited fractions of variation in community biomass when compared to taxonomic richness. Taxonomic richness was tightly related with the number of functional trait groups (total functional richness), the number of traits that can determine species’ responses to environmental changes (functional *response* richness), and the number of traits that modulate species’ effects on biomass (functional *effect* richness). On the other hand, the combined variation of taxon-specific abundances—which was driven by large canopy-forming autotrophs—was retained in the generalised additive model for community biomass. Both, functional richness and taxonomic composition accounted for significant fractions of the variation in community biomass, but the latter fraction was five-fold larger than the former (i.e. 51% and 10%, respectively). These results suggest that, in comparison to the environmental stress gradients here investigated, biodiversity might account for the largest proportion of variation in community biomass in Fildes Bay. Biodiversity, by providing key functional traits, may represent a high insurance value for the functioning of these subtidal Antarctic communities facing current and projected climate change scenarios.

### Spatial variability of taxonomic richness and community biomass

The diversity survey reported in our study varied in relation to similar studies carried in King George Island, e.g. in the sheltered areas of Admiralty Bay. In Admiralty Bay, macro-fauna at depths >10 m is dominated by filter feeders adapted to withstand sedimentation such as the bivalve *Laternula eliptica* and ascidians; in shallower sites, which are subjected to ice perturbation, mobile organisms such as *Serolis polita* are commonly found [31]. Similarly, Johnston et al. [47], reported marked differences in benthic composition between sheltered bays and islands in the East coast of Antarctica. While sponges and hydroids show high abundances in bays (likely due to ice and sedimentation regimes), canopy-forming algae such as *Desmarestia* sp. (6–12 m) and *H*. *grandifolius* (depth > 12 m) dominate the assemblages around the islands. On the other hand, the patterns described in our study are well in line with the spatial patterns of subtidal seaweed-dominated assemblages in the western Antarctic Peninsula [48,49]. Collectively, these results highlight the high scale-dependent spatial variation in subtidal assemblages in maritime and peninsular Antarctica.

In terms of abundance, brown algae followed by red algae were the dominant organisms accounting for more than 80% of the total biomass, especially at shallower locations. In the case of Rhodophyta, their increase in sites close to glaciers suggests advantages related with light use efficiency. In fact, many red algae living at depth >20 m normally are understory species shaded by the canopy of large brown algae of the order Desmarestiales. Another finding was that size of organisms, especially seaweeds, decreased with depth and closeness to glaciers, which could be related with a prevalence of sedimentary, more unconsolidated substrate at these locations and lower primary productivity due probably to enhanced light attenuation. Recently it was demonstrated that light conditions near glaciers, primarily characterised by enhanced melting and sedimentary runoff, limit algal photosynthesis and modify the lower limit of colonization of macroalgae [50]. Similarly, relative contribution of suspension feeders increased with depth and closeness to glaciers. In contrast to the patterns found in sedimentary bottoms [31], in Fildes Bay—which is characterised mostly by hard substrate—mobile organisms were less represented and not varied strongly across the environmental gradients. It should be born in mind, however, that the abundance of small organisms such as seaweed-associated amphipods and substrate-associated gastropods would have been underestimated in our study. Nevertheless, the complex and dynamic substrate characteristics of this zone may stimulate the partitioning of niches in the macrobenthic assemblage (see below), as has been previously suggested for the inner part of Fildes Bay [32].

### Relationships of functional richness and community biomass with taxonomic richness

In this study, we reported positive and saturating relationships between the number of taxa and functional groups of subtidal marine organisms. Albeit correlative patterns do not imply causality, these relationships might point to niche partitioning and a certain amount of insurance for functionality against species loss [11,51]. Nevertheless, the small fraction of variance in community biomass explained by functional richness suggests that niche partitioning has a relatively limited role in the assembly of these Antarctic subtidal assemblages. Moreover, functional response richness reached a saturating level at communities with relatively low taxonomic richness (ca. 10 taxonomic units), indicating that above this level higher taxonomic richness could not lead to increased functionality. Recent observational studies suggest that local-scale relationships between species richness and biomass depend on the environmental conditions, likely due to context-dependent productivity and strength of competition [18]. Probably, the strong environmental filters that characterise the subtidal ecosystem at Fildes Bay (e.g. [27]) may exert an overall control on the development of the assemblage and constrain the number of functional traits. For example, light limitation at depth close to 30 m (the depth at which penetrates the 1% of the surface light) defines different morpho-functional adaptations of macroalgal assemblages: massive and thick structures provide large brown algae with low light transmittance, while increased concentrations of light-harvesting pigments provide delicate red algae with increased abilities to absorb light in deeper waters [52].

While niche complementarity (here represented by the number of functional groups) accounted for a 10% of variation in community biomass, compositional effects accounted for more than 50% of the variation in this ecosystem function. Positive selection effects are evident when species’ contribution to community biomass and competitive abilities are positively correlated [53]. In our study, larger and massive primary producers were dominant across the system, accounting for a significant proportion of the community biomass. Dominant kelp-like algae, such as *H*. *grandifolius*, can monopolise the primary substratum and exclude competitively other benthic organisms (e.g. [54]), which can prevent the occurrence of resource complementarity among taxa (e.g. [55]). However, the interaction between taxonomic composition and functional diversity still accounted for a 25% of the variation in community biomass, suggesting that both, species composition and diversity can control the productivity of this system. The presence of key species with large effects on biomass and increased differences in functional traits, therefore, could interactively influence the richness-biomass relationship in these Antarctic communities. Further manipulative biodiversity-ecosystem functioning research should test this hypothesis in this system.

Besides largely contributing to community biomass, large autotrophs can also enhance the productivity of other species. Large, bioengineering autotrophs usually modify the environment and provide shelter to other smaller species in intertidal and subtidal habitats (e.g. [56–58]). Accordingly, the dominant kelp-like macroalgae could well have influenced the biomass patterns of the communities by facilitating the establishment and biomass accrual of other species, as previously shown between seaweeds and mesograzers in the western Antarctic Peninsula [59]. Many Antarctic brown algae show broad depth distribution between 5 and 30 m, which is mostly the result of low-light adaptation [60]. This characteristic of canopy-forming species provides the understorey with relatively stable environmental conditions along a wide depth profile [8]. The relationships between biodiversity (i.e. taxonomic richness and composition) and productivity can be bidirectional, as changes in the former can be both a cause and a consequence of changes in the latter [61]. Therefore, we suggest the hypothesis that facilitative interactions—mediated by species with key functional traits—could lead to bidirectional biodiversity-ecosystem functioning relationships and complex patterns of biomass, as those reported by previous observational studies in other ecosystems (e.g. [18,62,63]).

In summary, our study provided observational evidence of a consistent relationship between taxonomic richness and biomass of subtidal communities in Fildes Bay, west Antarctic Peninsula. Richness showed, in comparison with environmental stress gradients, a strong relationship with community biomass. Albeit this is an snapshot of the system, our results agree with previous manipulative work that supports the major role of biodiversity in the functioning of natural ecosystems [2]. The abundance of taxa with key functional traits, and resource complementarity in lesser extent, seemed to drive the biomass patterns. With this work, we hope to stimulate further manipulative research aimed to test the roles of key functional traits and resource complementarity in maintaining the functioning of marine Antarctic ecosystems.

## Supporting Information

S1 Data Entire dataset of wet weights of subtidal macrobenthic species at Fildes Bay, King George Island, Antarctica (csv format). (CSV)
